# Effects of conventional and ionic liquid-based surfactants and sodium tetraborate on interfacial tension of acidic crude oil

**DOI:** 10.1038/s41598-024-52178-1

**Published:** 2024-01-31

**Authors:** Mohammad Barari, Mostafa Lashkarbolooki, Reza Abedini, Ali Zeinolabedini Hezave

**Affiliations:** 1 Enhanced Oil Recovery (EOR) and Gas Processing Research Laboratory, Faculty of Chemical Engineering, Babol Noshirvani University of Technology, Babol, Iran; 2 Department of Management, Bahonar, Fars Branch, Technical and Vocational University, Tehran, Iran; 3 Arak Science and Technology Park, Fanavari Atiyeh Pouyandegan Exir Company, Arak, Iran

**Keywords:** Chemical engineering, Chemistry, Energy science and technology

## Abstract

The application of a new class of surfactants such as ionic liquids (ILs) compared with the conventional surfactants and their interactions with each other concomitant and alkaline under salinities is not well examined based on the best knowledge of the authors. So, the current work focused on the impact of sodium lauryl sulfate (SDS), sodium dodecyl benzene sulfonate (SDBS), 1-dodecyl 3-methyl imidazolium chloride (C12mim][Cl]), 1-octadecyl 3-methyl imidazolium chloride ([C18mim][Cl]) in the presence and absence of alkali namely sodium tetraborate known as borax (Na2B4O7) on the IFT variation while the salinity was changed 0–82,000 ppm (ionic strength of 0–1.4 M). The results showed the positive impact of salinity on the pH reduction and reduced the alkaline effect for pH reduction. Also, the measurements showed that the presence of surfactant reduces the role of alkaline for pH variation as it moved from 9.2 to 6.63 for the solution prepared using SLS and SDBS. The measured IFT values showed that not only alkali has a significant impact as it combined with SLS and SDBS due to a desired synergy between these chemicals, it can reduce the critical micelle concentration (CMC) for the SDBS from 1105 to 852 ppm and much higher for [C12mim][Cl].

## Introduction

Due to several complexities, a proper amount of the trapped oil can be recovered using the reservoir's natural pressure. On the other side, the oil production using the reservoir's natural pressure is insufficient for the current energy consumption and considering the growing rate of population. So, considering these limitations and concerns concomitant with limited available oil reserves in the world, moved the researchers toward methods commonly known as enhanced oil recovery (EOR) and improved oil recovery (IOR) techniques to produce a higher rate of trapped oil in the reservoir^1–3^.

According to the different possible approaches in the field of EOR processes, these methods can be categorized into chemical injection known as chemical EOR (CEOR)^4–9^, thermal-based methods, low salinity or smart (engineered ion) water injection, carbon dioxide or dissolved carbonated in water (carbonated water) injection, etc.^10–12^. Among the different possible EOR methods, using chemical injection (alkali, surfactant, and polymer) is of great interest, especially if the oil price is high. This method and chemicals are highly desired since they can manipulate multiple mechanisms such as surface phenomena of interfacial tension and rock/fluid interactions known as surface wettability^13–22^.

In this way, various chemicals are used in CEOR such as surfactants^23^, ion-engineered water^24^, alkalis and polymers^25,26^, and nanoparticles (NPs)^27^. Among these chemicals, surfactants and nanoparticles are used in different industries due to the direct impact on the interfacial tension and surface wettability^28^.

For example, Kumar et al.^29^ investigated the impact of surfactant/nanoparticle on the tertiary oil recovery using a core flooding experiment and even a simulation approach. The point is that they reported that the initial IFT value of 63 mN/m was reduced to the value of 0.12 mN/m as the concentration of Tween 40 surfactant was increased to 0.5 wt %.

They also reported that ultra-low interfacial tension (IFT) reduction ability, low dosage self-aggregation property, high values of solubilization parameters, and enhanced miscibility property of non-ionic surfactant (Tween 40) are the main reasons for producing stable Nano-emulsions. The interesting point that Kumar et al.^30^ mentioned is that the IFT was also further reduced from 0.17 mN/m to 0.007 mN/m as the SiO2 NPs concentration was increased from 0.0 wt % to 0.03 wt %. This reduction in IFT is attributed to the favorable adsorption of NPs at the oil–water interface and a decrease in interfacial energy barrier^31–33^.

Moreover, Ramezani et al.^34^ and Fatahhi et al.^35^ examined the impact of SiO2-Nps on the IFT reduction and wettability alteration. Ramezani et al.^34^ reported that the effect of used NPs such as SiO2-NPs is directly related to the nature and type of the used surfactant. For example, they reported that using SiO2-NPs concomitant with TiO2-surfactant has a slight effect on eth IFT reduction by reducing the IFT from 2.8 to 1.5 mN/m while using SiO2-NPs with sodium lauryl sulfate (LSL) has a profound effect on eth IFT reduction (0.36 mN/m to 0.1 mN/m).

On the other side, Fatahhi et al.^35^ reported that although the addition of SiO2-NPs has a profound impact on the wettability alteration from neutral-wet to strongly water-wet, it has a slight effect on the IFT reduction similar to the results reported by Ramezani et al.^34^ obtained for pyridinium based ionic liquid and TiO2-surfactant. However, the results obtained for SLS) were completely different leading to wettability alteration from moderate water-wet to rather strongly water-wet condition and IFT reduction from 0.36 to 0.1 mN/m.

Among the different chemical methods, alkaline and surfactant injection under controlled salinity conditions are highly applicable for higher oil production. Unfortunately, since using only one chemical is inefficient to be used for ultimate impact on the oil recovery by activating multiple mechanisms, it is recommended to use a combination of alkali-surfactant, alkali-polymer, or surfactant-polymer, etc. for better efficiency during chemical injection. On the other side, since the reservoir conditions are highly complicated new approaches that use different EOR methods together, known as the hybrid method, seem more applicable. The concept behind this combination comes from mixing the impact of each method achieving ultimate oil recovery of the trapped oil by activating multiple mechanisms and providing better synergy between different chemicals. Among the possible chemical combinations, combining alkaline with surfactant under controlled salinity conditions seems applicable for higher oil production due to several advantages.

In general, surfactants are categorized into non-ionic, anionic, cationic, and zwitterionic categories^36^ considering their chain and tail structure including hydrophilic and hydrophobic types^37,38^ which are mainly comprised of polymeric or hydrocarbon type chains which directly dictate the surfactant nature^39^. Besides the surfactant molecules, alkali can be used in the CEOR processes to form in-situ surfactants and acts as a sacrifice for surfactant by being adsorbed in the first place, consequently reducing the surfactant adsorption toward more economic condition. For example, Saha et al.^40^ examined the possible relation between IFT and wettability during the saponification process occurring in the presence of alkali such as sodium hydroxide (NaOH) for different crude oils of light and medium types via core flooding experiments. They found that the presence of NaOH has a considerable reverse impact on the IFT reduction as the alkali concentration increases and extracts the residual oil from the pore networks of the reservoir.

Moreover, Sun and his coworkers^41^ examined the crude oil composition influence on alkaline, polymer, and surfactant chemicals through alkaline (sodium hydroxide)-surfactant (alkyl benzene sulphonate)-polymer (partially hydrolyzed polyacrylamide) (ASP) combination. Sun et al.^41^ reported that the oil/ASP slug IFT was more desired than those measured for another type of examined oil. They correlate the observed trend to the effect of dissolved active chemicals in the oil phase left the oil/water interface. Also, Cai et al.^42^ examined the IFT of several alkane/brine systems for pressures and temperatures of 0.1–30 MPa and 298.15–353.15 K, respectively, which showed a linear relationship for pressure and salt concentration with a slight effect of salt type on the IFT. Similar to the undeniable impact of alkali on the IFT reduction, especially for the highly acidic crude oils, mono and divalent salts are also highly effective on the IFT reduction although their impact is not well understood. For example, Nasr-El-Din and Taylor^43^ investigated the Neodol 25-3s and Triton X-100/ enhanced alkaline/Lloydminster crude oil IFT value interestingly revealed that there is no generalized rule regarding the impact of surfactants on the IFT reduction under high salt condition.

Furthermore, Rudin et al.^44^ examined the IFT variation for the alkaline/surfactant/acidic oils systems. They claimed that ultra-low values are possible if acidic contents of crude oil are ionized and form mixed micelles. They also mentioned that surfactant molecules retard the mass transfer and increase the equilibrium time of IFT.

Among the recently performed investigations, Saw and his coworkers^45^ performed an investigation regarding the possible synergy between two nonionic surfactants of Tergitol 15-S-12 and PEG 600 considering IFT and wettability alteration concomitant with the impact of optimum chemical formulations on the oil recovery using core flooding experiments as the main parameters. They reported that using Tergitol 15-S-12 and 1 wt % of PEG 600 led to an optimum chemical formulation capable of reducing the IFT of a mixture (aqueous solution/crude oil) to an ultra-low IFT value of 0.672 mN/m. Moreover, they reported that the optimum chemical formulation capable of changing the wettability toward water-wet condition concomitant with its capability to lower the IFT values led to 17.73% oil recovery based on original oil in place (OOIP).

In another investigation to enlighten the impact of surfactants on the different effective parameters besides the IFT reduction, Kumar and Mandal^46^ investigated the effect of zwitterionic surfactant on the adsorption and wettability alteration using two rocks of sandstone and carbonate types.

They reported that the examined surfactant was highly capable of changing the wettability of the rocks toward more desired conditions leading to higher oil recovery in the light of surfactant adsorption on the rock surface. Moreover, they reported that besides the surfactant type, salinity and alkalinity are the other crucial and effective parameters on the surfactant adsorption and wettability alteration. They reported that the presence of alkali decreases the contact angle of the drop of the sample due to the generation of OH ions and carbonic acid on the dissolution of Na2CO3 in water. In detail, as the OH ions form, in-situ surfactant molecules appear to increase the hydrophilicity of the surface of the sample leading to better water-wet conditions^40^.

According to these findings, it can be concluded that in the case of using alkaline in the solutions, two parameters of the rock wettability and further IFT reduction if it uses concomitant with surfactant. In this way, it seems that investigating the effect of alkaline in the optimum chemical formulation through the surfactant flooding is highly important whether its impact on the IFT or wettability is investigated. Also, Zhao et al. reported that dynamic IFT (DIFT) of aqueous solution/ acidic crude oil can experience ultra-low IFT values even if a low surfactant concentration is dissolved in the solution. Their findings showed the significance of salt on the surfactant adsorption onto the interface and phase partitioning. Unfortunately, it is well established that both pH and acidic contents have a significant impact on the IFT although there is no clear correlation between these two parameters and IFT reduction. For example, several researchers reported both increasing and decreasing effects of IFT at low pH values and each of these studies correlated this observed trend to resin and asphaltene fractions^47–49^.

Besides the conventional surfactants, a new class of surfactants has been under investigation during the past two decades from the ionic liquids (ILs) family. In general, ionic liquids (ILs) are known as salts with melting or glass transition temperatures below 100 °C which is low enough that put most of the ILs at the liquids phase under room temperature, which means easy handling and application of these chemicals. Moreover, since they have negligible vapor pressure concomitant with high thermal stability and solvating capacity, they are a good candidate for EOR purposes^50^, Hezave et al.^51–54^. The other advantage of the ILs puts them at the top of the list of researchers is their tunability. In detail, since it is possible to use different cations and anions to produce and fabricate different ILs that make them task-specific compounds which can be easily designed concerning the characteristics of each reservoir^55,56^.

In this way, Zabihi et al.^6^ examined conventional (sodium dodecylbenzene sulfonate (SDBS)) and IL-based (imidazolium and pyridinium chlorides) surfactants as two different classes of surfactants. They reported that both of the examined surfactants were capable of tolerating harsh salinity conditions up to 50,000 ppm although using SDBS concentration higher than 1600 ppm led to fast precipitation in the aqueous solution while the examined [C12mim][Cl] and [C18mim][Cl] showed the best IFT reduction capability without precipitation.

Sakthivel and his coworkers^57^ examined the effect of SDS and several alkyl ammonium ILs under different salinities and temperatures for possible application through oil recovery processes. Among the examined surfactants, [OHPrNH3][CF3COO] revealed the best outcome for tertiary oil recovery purposes by reducing the IFT values to a value of 9.38 mN/m which is not an ultra-low value. The performed core flooding experiments using sand packs revealed that combining the ILs with the polymer during core flooding stages led to a maximum oil recovery of 17–23% based on the OOIP using low salinity conditions. As the last point, they pointed out that the other effective parameter that guarantees the efficiency of the used ILs is their alkyl chain length as the alkyl chain increases, the higher the oil recovery means.

Year after, Sakthivel and his coworkers^58^ examined the possibility of tertiary oil recovery using lactam and imidazolium-based ionic liquids (ILs) under high salinity conditions. Their findings demonstrated that using [CP][C6H13COO] led to the best EOR performances, especially under high salinity conditions. Similar to the previous study of Sakthivel and his coworkers^57^, although the IFT values were higher than 8 mN/m, the core flooding experiments revealed the possibility of increasing the oil recovery in the range of 22–25% based on OOIP and the oil recovery was increased up to 31% based OOIP if the salinity was changed to high salinity conditions.

Furthermore, Somoza et al.^59^ investigated the effect of [C10mim][OTf] on the IFT of crude oil/aqueous solution. They reported that increasing the concentration of IL in the range of 2000, 3000, 4000, and 5000 ppm led to IFT reduction to values of 6.70, 2.42, 0.36, and 0.41 mN/m, respectively, using distilled water. They also reported that as the concentration of NaCl was increased from 0.1 wt% to 1 wt%, the IFT value would increase from 0.36 mN/m to 0.57 mN/m and 0.93 mN/m and further increase in the NaCl concentration to values of 2 and 4 wt% had no significant effect on the IFT regardless of increasing or decreasing trend.

Respecting these points, the current work which is the complementary phase of the previously published literature^60–62^ is aimed to investigate the synergy between the four chemical surfactants from the conventional family (anionic surfactants namely SDBS, sodium lauryl sulfate (SDS)), IL family (1-octadecyl-3-methyl imidazolium chloride ([C18mim][Cl]) and 1-dodecyl-3 methyl imidazolium chloride ([C12mim][Cl])) and one alkaline namely sodium tetraborate commonly known as borax (Na2B4O7). In the investigation performed by Ramezani et al.^62^, the results revealed that tuning ions composition of SO − 4, Ca2 + , and Mg2 + is a vital parameter through the oil recovery processes besides the oil composition (particularly asphaltene and rein fractions). They reported that the structural properties of crude oil fractions, especially asphaltene and resin fractions with the ion composition (ionic strength = 0.8 M which is smart water) and a new class of surfactant which was 1-dodecyl 3-methyl imidazolium chloride (C12mim][Cl) had undeniable effects on the IFT variation including the IFT value and adsorption times of these two fractions.

Moreover, Barari et al.^60,61^ performed investigations to find the effect of the alkyl chain length of two different ILs of [C12mim][Cl] and [C18mim][Cl] in the presence of Na2SO4 and NaCl on the surface properties such as wettability and IFT. They reported that both ILs with the examined salts changed the wettability toward the water-wet conditions with a minimum contact angle of 34o and minimum IFT of 0.5 if the ionic strength values were changed between 0–1.4. They also reported that among the different examined salts it seems that the application of salts with sulfate anion is more effective for IFT reduction since they can lose the lateral compression between the tails of the examined ILs.

According to these facts regarding the profound effect of salinity on the interactions between different chemicals in the reservoirs, the salinity was tuned in the range of 0–82,000 ppm (ionic strength of 0, 0.07, 0.7, and 1.4 M) using two different salts of sodium sulfate (Na2SO4) and sodium chloride (NaCl). The other point is that in contrast to the previously performed literature, the current investigation not only concentrated on the individual impact of these chemicals on the IFT reduction but also the combinative impact of these chemicals under binary and ternary conditions were examined for the first time. In detail, since the synergy between the surfactants can introduce desired implications on the IFT reduction with lower concentrations of chemicals especially if they combine with the alkaline which can act as the sacrifice and in-situ surfactant producer, examining this combination is highly important for industrial purposes where the synergy can control the operating cost. In other words, the current work is examining the effects of five chemicals including four surfactants and one alkaline in individual conditions, binary and ternary mixtures for the first time.

## Materials and methods

### Formation brine

In the current study, two types of base aqueous solutions were prepared one of them is purely distilled water (DW), and the second one is the aqueous solution prepared by dissolving NaCl in the range of 0–82,000 ppm changing the ionic strength of the aqueous solution in the range of 0–1.4 M. One of the synthetic brine solutions (SW) was prepared with ionic strength of 0.7 which is similar to the ionic strength of the Persian Gulf seawater using the following Eq. 1:

1 $$ I = \frac{1}{2}\sum\limits_{i = 1}^{n} {C_{i} Z_{i}^{2} } $$

where Zi is the valence of ionic species, and Ci is the concentration of salt.

### Synthesis procedure of ionic liquids

Diethyl ether was reacted with 1-methylimidazole, and 1-chlorododecyl with a purity better than 99.9% (Merck/Fluka) to prepare 1-dodecyl-3-methylimidazolium chloride ([C12mim]Cl) and 1- octadecyl-3-methylimidazolium chloride ([C18mim]Cl). In the first step, an excess amount of 1- chlorododecane was placed in a bottle with 1-methylimidazolium with good agitation. Secondly, the resultant solution was moved to a heated flask (temperature of 70 oC) and stirred for two to three days. The point is that the flask was equipped with a reflux assembly and the system was heated to the point that no solvent remained. The resultant solution at this point is a thick, viscous, and yellowish solution that must be washed for more than 5 cycles using diethyl ether to reach the purest version of the IL. Finally, a dryer (an oven at a temperature of 100 oC) was used to remove any solvent from the resultant IL. The wavelength results (2800–3000 cm^−1^) of FT-IR in the was correlated to the stretching vibration of CH, CH2, and CH3 similar to those reported previously^63,64^. Besides, C-N and C = C binding for the imidazolium ring can be detected with values of 1062 and 1560 cm − 1, respectively by an investigation of the wavelength results. Also, investigating the FT-IR analysis revealed the presence of aromatic CH and CH2 stretching attributed to the imidazole ring with peaks of 780 and 1462 cm − 1, while the stretching vibration for O–H was observed at the wavelength of 3440 cm^−1^^63–65^.

### Crude oil

The sample oil was kindly supplied by the National Iranian South Oil Company (NISOC) from one of the southern Iranian oilfields and has a density of °API of 32.28 @ room temperature with about 7.53% and 10.09%, asphaltene and resin contents, respectively. FTIR analysis was used to find more information about the asphaltene and resin fractions. Analyzing these two fractions is necessary since these two fractions besides saturates and aromatics can introduce profound effects on the surface phenomena including IFT. According to the obtained FTIR spectra, it is possible to see that the peak of O–H stretching of alcohol at 3400–3600 cm^−1^, stretching vibration of C = O for ester at 1738 cm^−1^, stretching of S = O for sulfonyl chloride at 1372 cm^−1^, stretching of C–O for carboxylic acid at 1217 cm^−1^, respectively. However, the aliphatic cycloalkanes and hydrocarbons C–H stretching and –CH2– stretching were observed at 2800, and 2999 cm^−1^^64–67^. The point is that besides the C–O bond observed at 1012 cm^-1^, 1445–1485 cm^-1^ wavelength range is an indication of CH2, and 1590–1610 cm^-1^ wavelength is correlated to the CH3 asymmetric deformation, and C = C stretching in aromatic rings, respectively^64^. Moreover, N–H bonding for the amide functional group and C–H stretching for aromatic hydrocarbons were observed for the wavelength range between 700 and 900 cm^-1^^64–67^.

### Interfacial tension measurement procedure

Use of the pendant drop method for IFT measurement is increasing due to its high accuracy and reliability besides its capability to be used under high temperature-high pressure conditions^68^ (APEX Technologies Co., Arak, Iran)). This method is well-known not only due to its accuracy and simplicity but also due to its capability to be performed under high pressure-high temperature (HP-HT) conditions. This method mainly includes different sections of a) injecting drop at the tip of the nozzle to form a suspended drop for shape analysis purposes, b) an image capturing system of a CCD camera coupled with a macro lens to provide a proper size of the formed image at the tip of the nozzle, and c) an image processing software (preferably on-line which also can be off-line). A detailed description of using this method is given elsewhere which is theoretically based on the following equation (Eq. 2):

2 $$\gamma =\frac{\Delta \rho g{ D}^{2}}{H}$$

where *g* is the gravity acceleration, *Δρ* is the difference of bulk and drop phase density and *H* is the shape-dependent factor. Considering the equatorial diameter (*D*) and the distance *D* from the top of the drop known as *d*, one can calculate the shape factor by dividing d by *D (S* = *d/D)*. The maximum uncertainty of the measured IFT values was ± 0.2 mN/m which was obtained by averaging at least three different independent measurements.

## Results and discussions

### Impact of chemicals on the pH of aqueous solution

**Figure 1:**
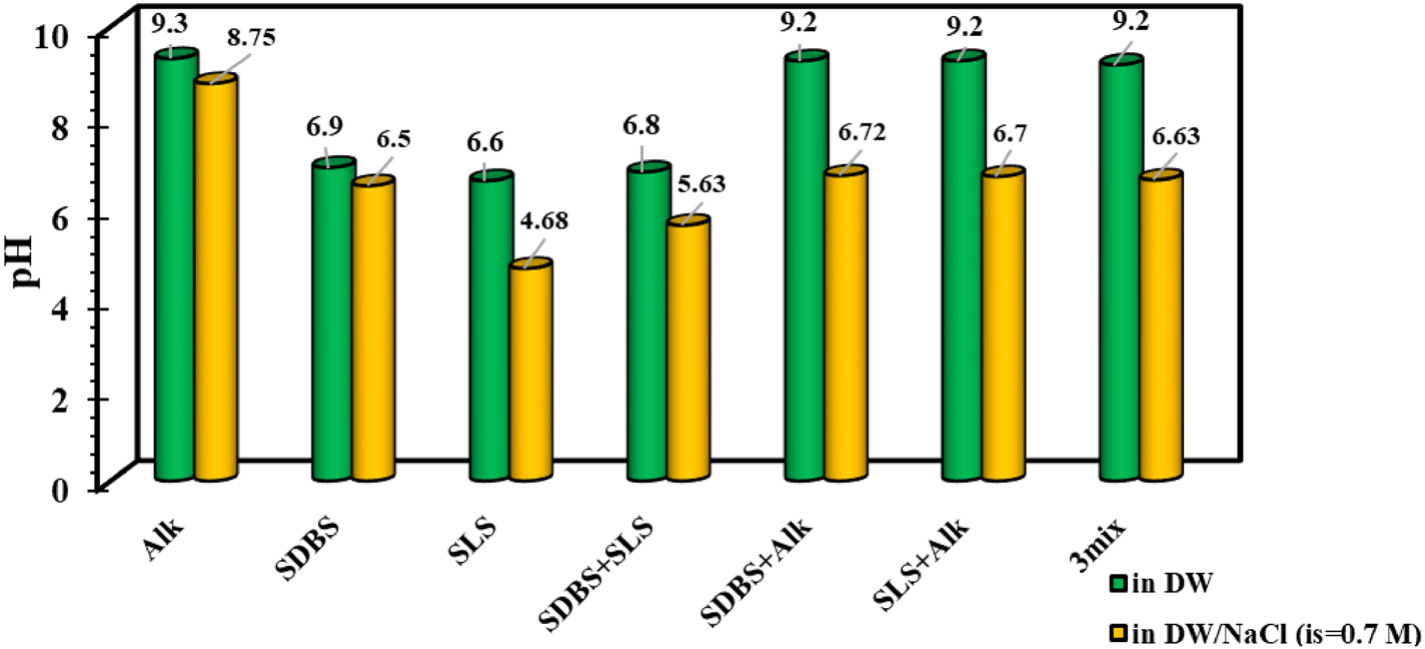
Effect of different chemicals on the pH of the aqueous solution at the absence and presence of salinity (ionic strength of 0.7 M).

In the first stage of this investigation, the effect of different chemicals on the pH variation was investigated using two aqueous solutions of DW and a modified solution with ionic strength of 0.7 M using NaCl. A glance into the results depicted in Fig. 1 revealed that dissolving 1000 ppm of alkali in the DW increases the pH to 9.2 similar to the solutions prepared by dissolving 500 ppm SDBS + 500 alkali, 500 ppm of SLS and 500 ppm of alkali or a ternary mixture of SDBS/SLS/alkali with similar concentration of 333.3 ppm. According to these findings, it seems that the effect of alkali in the solution is more dominant in controlling the pH of the solution than the surfactants.

**Figure 2:**
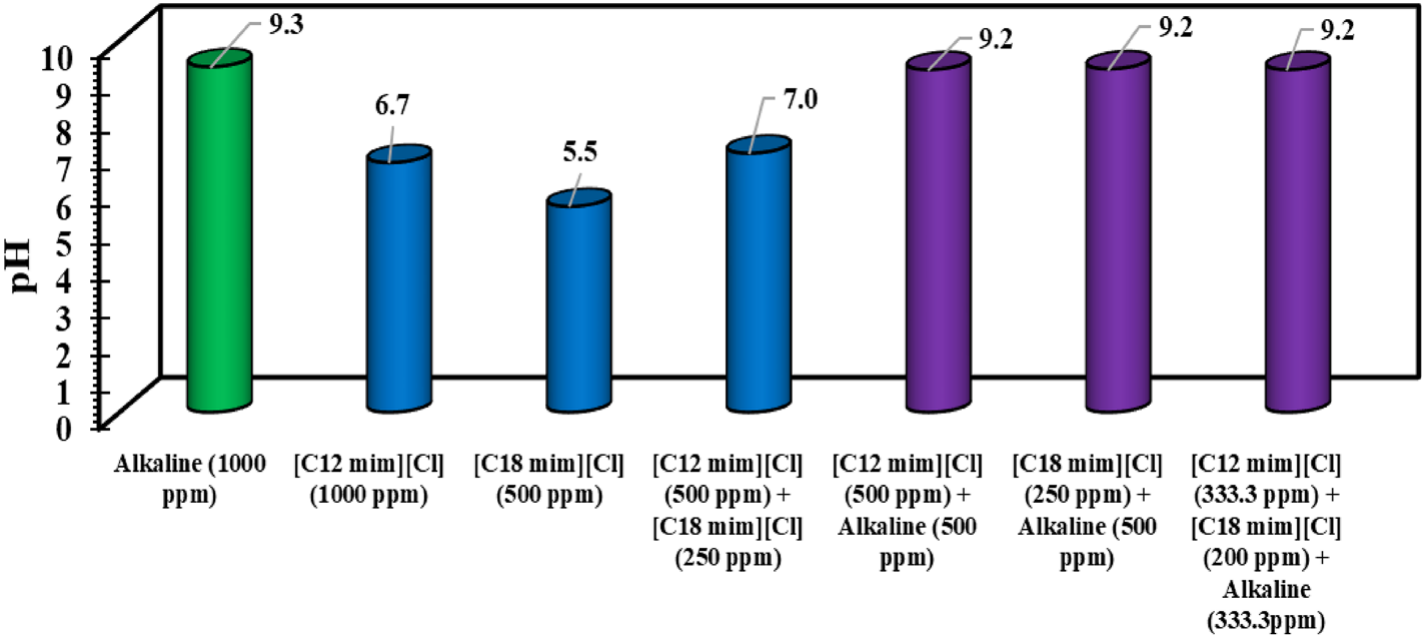
The pH variation as a function of ILs in the absence of salinity.

The other point is that the presence of salinity reduces the pH even for the alkali solutions. In detail, it seems that the presence of NaCl in the solution can reduce the ionization of the alkali and control the pH to some extent. Besides, comparing the results for the alkali solution modified by NaCl and the surfactant solutions modified by alkali and NaCl, one can conclude that the surfactants are activated for controlling the pH values toward lower values in the presence of NaCl. In other words, it seems that the NaCl ions can disperse in the solution and provide a better chance for the surfactant solutions to prevent the ionization of the alkali molecules for higher pH values. Moreover, the results in Fig. 2 revealed a similar trend for the examined ILs with slight deviations. According to these findings, it seems that the best scenario for the optimum chemical formulation is the application of distilled water modified by multiple chemicals of SDBS/SLS and alkali or [C12mim][Cl]/[C18mim][Cl]/alkali with an equal concentration of 333.3 ppm leading to the highest pH value which is highly necessary to produce in-situ surfactant during the saponification process. The point is that using distilled water to prepare the optimum chemical formulation is impossible due to technical limitations and considering the operating cost. In this way, the optimum chemical formulation is the mixture of surfactants or pure alkaline dissolved in the aqueous solution with an ionic strength of 0.7 M.

### Effect of different surfactants on the IFT

**Figure 3:**
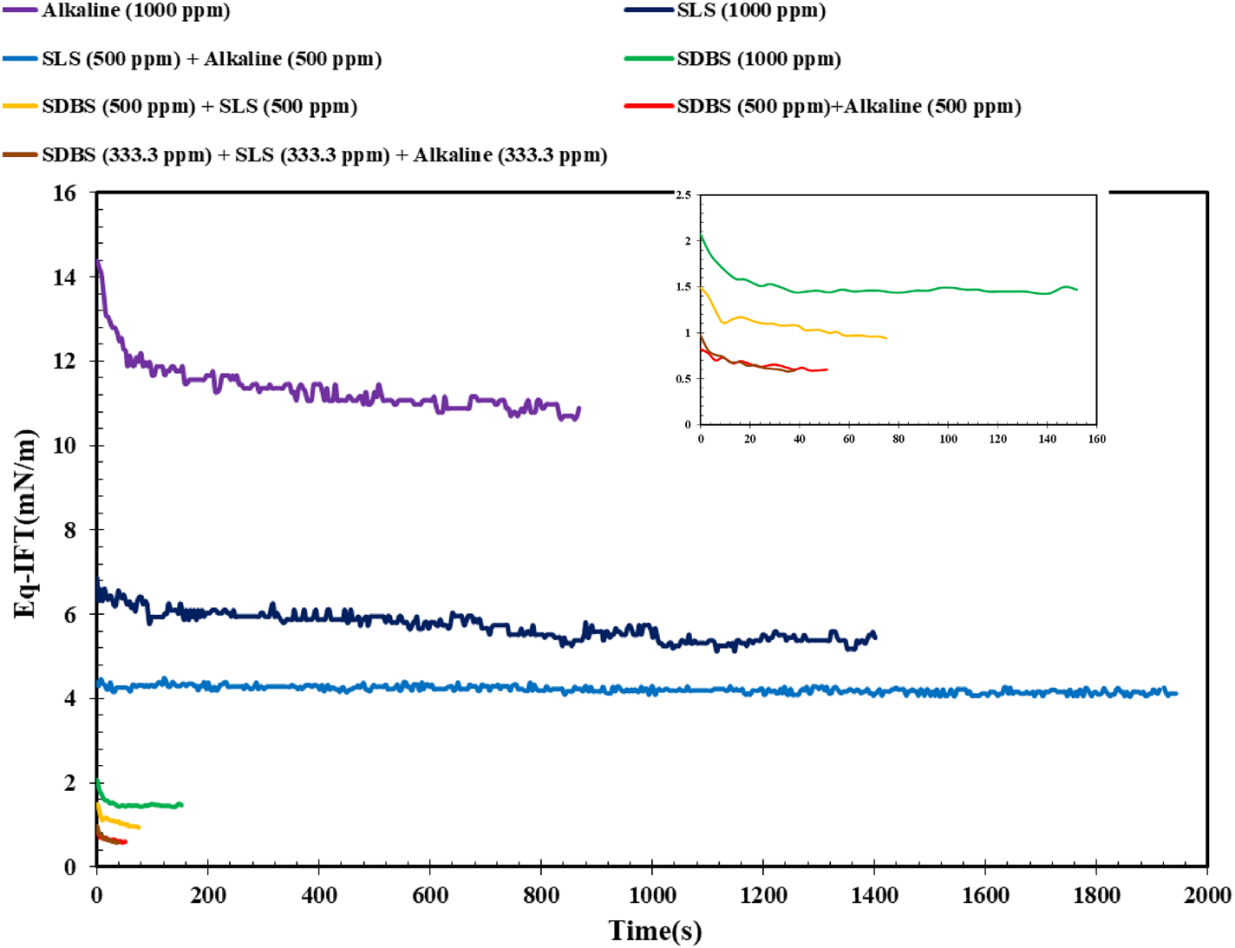
Effect of chemical formulations on the DIFT using DW.

In the next stage of this investigation, the effects of different chemical combinations were examined on the dynamic IFT (DIFT) using the aqueous solutions prepared by DW (see Fig. 3). A glance into Fig. 3 revealed that both SLS solutions (1000 ppm SLS and 500 ppm alkali + 500 ppm SLS), no dynamic behavior are obtained while for the alkali solution and the other examined chemical solutions, it is possible to see the dynamic behavior during the IFT measurements although several of these solutions have a narrow range of dynamic behavior. In detail, for the solutions SLS exists on them, only a straight line is obtained means no dynamic behavior for these solutions. However, there is an obvious reducing dynamic trend for IFT of the alkali solution dissolved in DW in contact with the acidic crude oil.

This finding is completely correlated to the formation of the in-situ surfactants that come from the saponification between the fatty acidic component of the crude oil and OH– of the alkali released in the aqueous solution. Since the reaction between these two compartments is time-consuming, the dynamic behavior is due to the formation of in-situ surfactant as a function of time which reaches a plateau after a while. A similar trend can be observed for the solutions prepared by pure SDBS (1000 ppm), SDBS (500 ppm) + alkali (500 ppm), SDBS (500 pm) + SLS (500 ppm), and SDBS (333.3 ppm) + SLS (333.3. ppm) + alkali (333.3. ppm). The reason behind these obtained results can be correlated to the structure of the SDBS having a long tail and sulfonate head group leading to the gradual orientation of the different molecules in the interface leading to fast equilibrium concomitant with dynamic behavior. In other words, since the SDBS structure is lengthy with the more active cationic head group, it can provide more sites to adsorb the different ions and chemicals in its structure causing a dynamic behavior. But, its highly active nature leads to fast equilibration of IFT or narrow time band for dynamic behavior. In total, it seems that among the examined chemical solutions, using SDBS (500 ppm) + alkali (500 ppm) leads to the lowest IFT value which means this formulation is the optimum formulation to reach the lowest IFT value.

**Figure 4:**
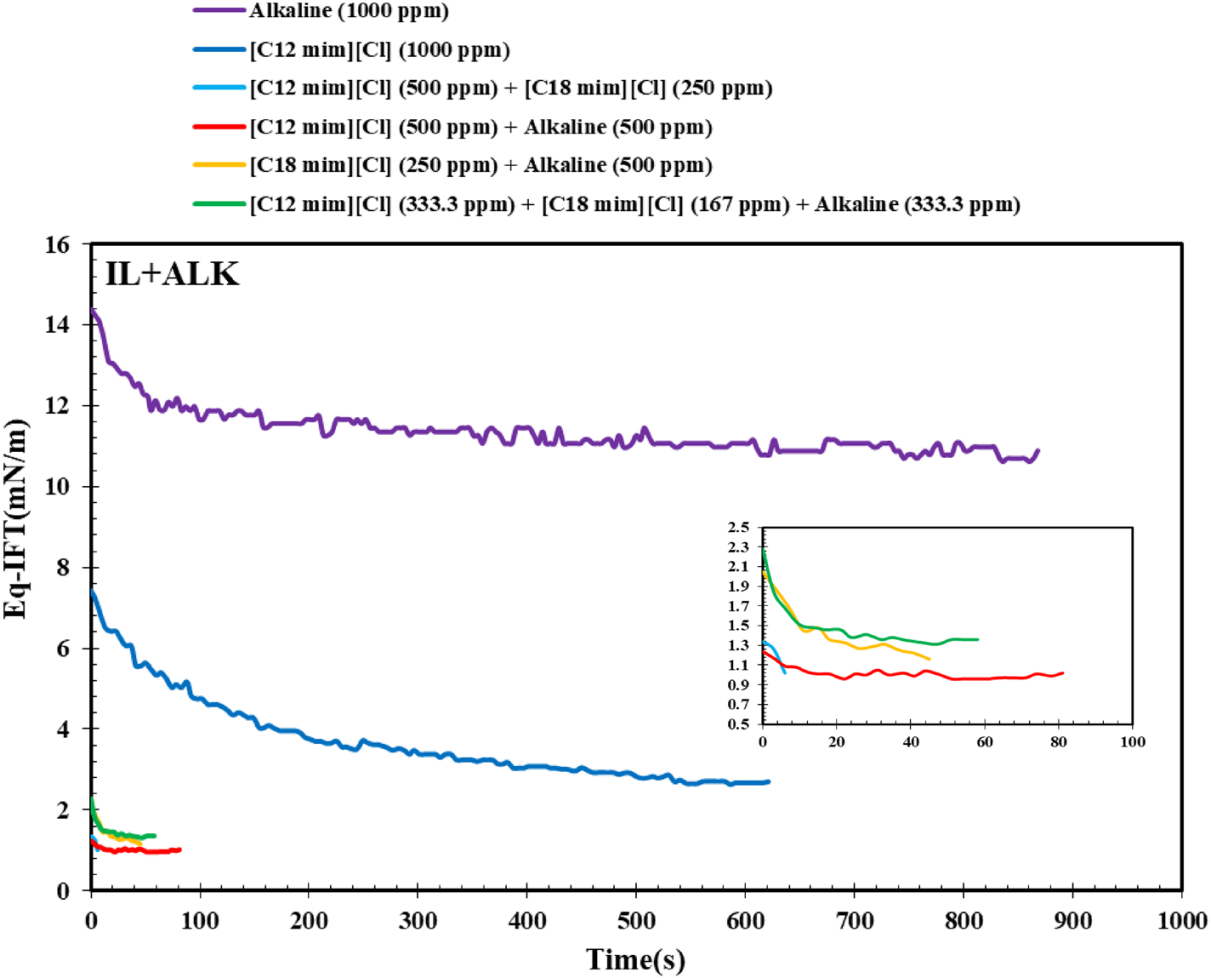
Effect of ILs on the DIFT using DW.

Besides, the effect of alkali on the IFT variation in the presence of different ILs was examined with a maximum overall concentration of 1000 ppm as it was previously performed for the conventional surfactants (see Fig. 4). A glance into Fig. 4 revealed that for all of the examined chemical formulations, dynamic behavior is unavoidable. This observed trend can be correlated to the formation of in-situ surfactants during the saponification processes concomitant with the presence of ILs. More investigations revealed that among the examined ILs, the shorter chain length one ([C12mim][Cl]) leads to better IFT reduction than the longer chain length IL ([C18mim][Cl]). The reason behind this observed trend can be correlated to the fact that the longer chain length leads to higher adsorption of the alkali molecules in its structure which in the first stage leads to a sharp reduction in IFT (see Fig. 4 (yellow line)) and then reaching a plateau due to supersaturation of the longer chain length sites leading to an increase in the repulsive forces consequently leading to reduce the IFT reduction rate and even stop it. On the other hand, combining the [C12mim][Cl] which has a shorter chain length than [C18mim][Cl] experienced a moderate but longer IFT reduction rate leading to a lower ultimate IFT value as demonstrated in Fig. 4 (red line).

### Critical micelle concentration (CMC) calculation

**Figure 5:**
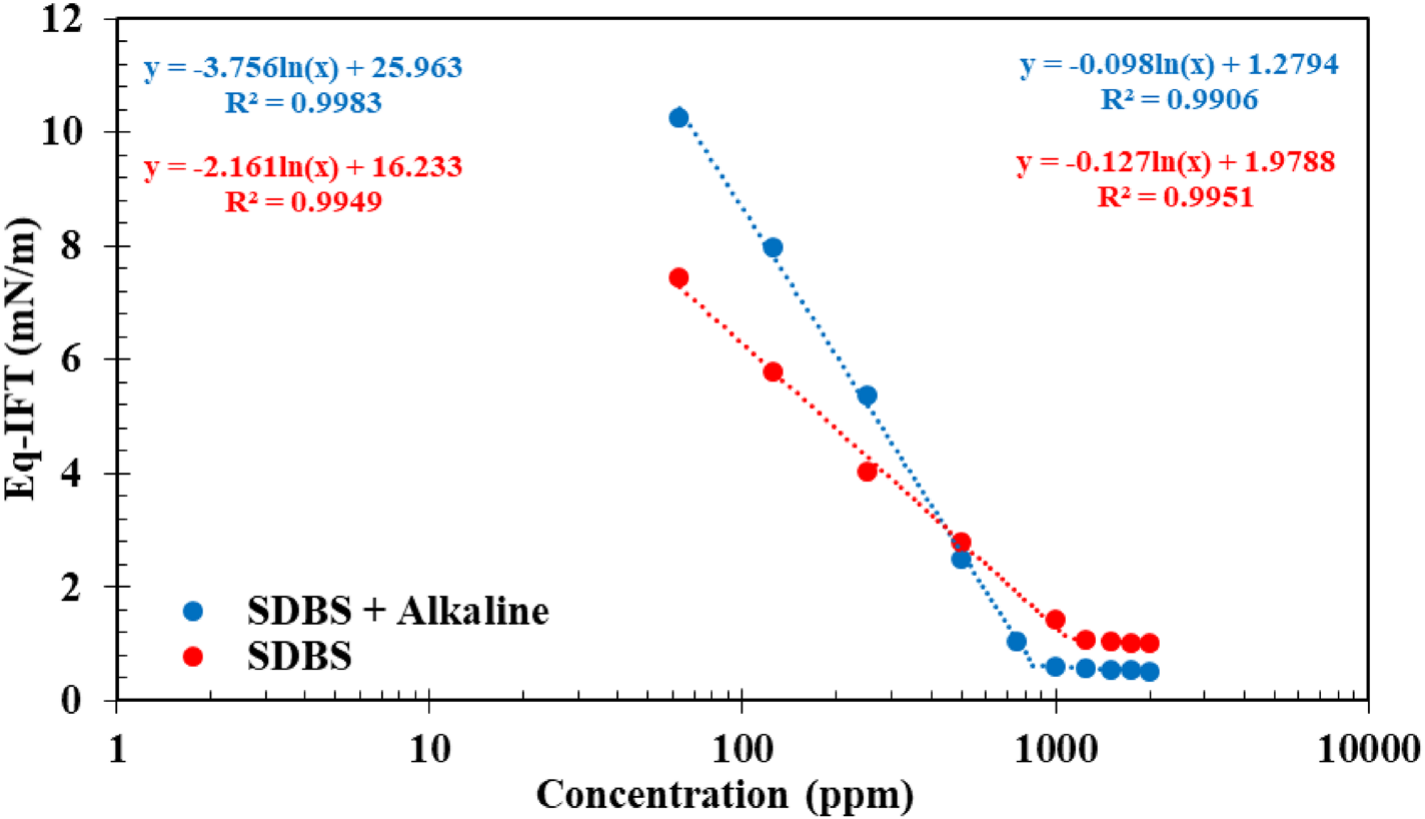
Impact of alkali on the CMC modification of SDBS.

**Figure 6:**
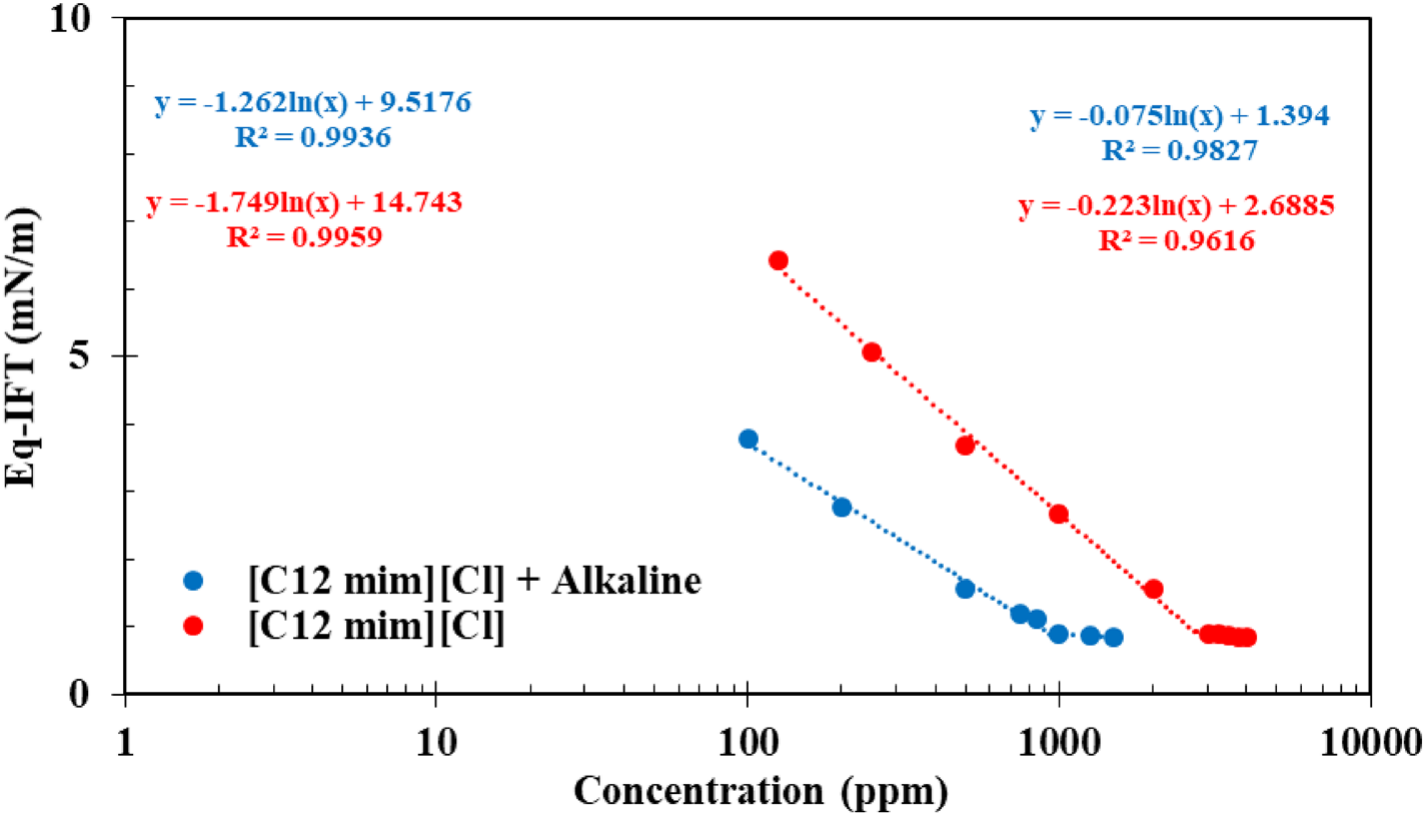
Impact of alkali on the CMC modification of SDBS.

In the next stage of this investigation, the impact of alkali on the CMC of the SDBS and [C12mim][Cl] was investigated using DW since these two surfactants concomitant with the alkali (500 ppm) leading to the lowest IFT values. A glance into the results depicted in Figs. 5 and 6 revealed that the presence of alkali in the aqueous solution led to a reduction in the CMC value for both SDBS and [C12mim][Cl] from 1105 to 852 ppm and 2696 to 938 ppm, respectively. According to these findings, one can conclude that the impact of alkali on the CMC reduction is more profound for [C12mim][Cl] than the SDBS surfactant. The reason for this observed trend is directly correlated to the structure of the IL compared with the SDBS leading to easier packing of the alkali molecules in its structure and lower repulsive forces leading to a sharper reduction in the CMC value of the IL surfactant.

### Effect of salinity on the IFT variation

**Figure 7:**
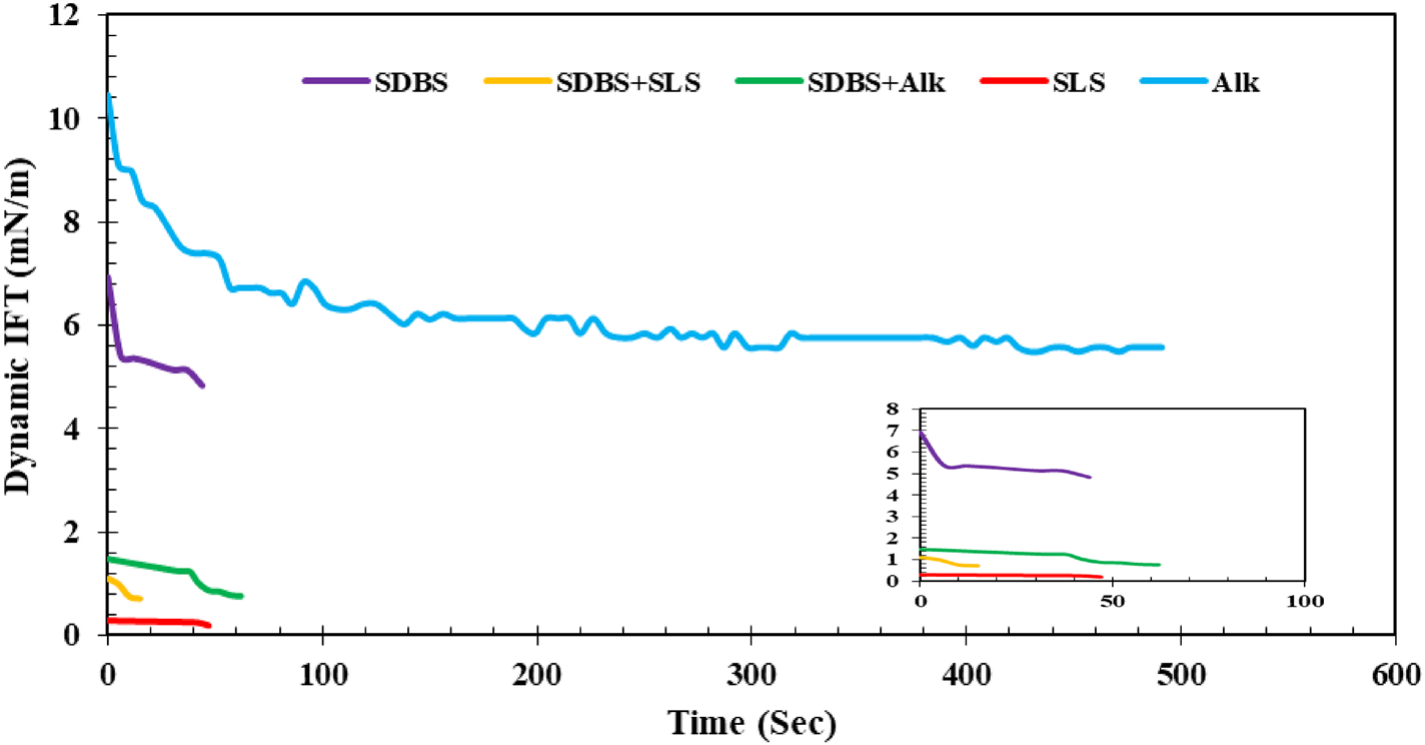
Effect of chemical formulations on the DIFT using saline water with ionic strength of 0.7 M using NaCl dissolution.

In the next phase, the effect of different chemicals on the IFT was investigated using a saline solution prepared with ionic strength of 0.7 M (see Fig. 7). The obtained results revealed that although the presence of salinity leads to a better reduction in IFT using only alkali in the solution, it keeps the dynamic behavior of the used alkali solution similar to the trend observed for the pure alkali in the absence of salinity. A closer look into the results revealed that in contrast to the results obtained for alkali, the presence of salinity significantly reduces the functionality of SDBS for IFT reduction due to salinity impact on the head group leading to degradation or precipitation of SDBS in the solution leading to higher IFT values for SDBS solution prepared using saline water. On the contrary, the results revealed the better efficiency of the SLS in the presence of salinity for IFT reduction while the capability of the other chemical combinations for IFT reduction remains constant. According to these findings, one can conclude that among the examined combinations, SDBS is not a good candidate for the solution that deals with harsh salinity conditions. Similar results were reported by Zabihi et al.^6^ regarding the precipitation of SDBS in salinities higher than 50,000 ppm. In detail, they reported that as the concentration of salts increases to a value higher than 50,000, the SDBS molecules leave the solution and collect as the precipitate at the bottom of the flask which makes it an improper surfactant for the system dealing with high salinity conditions. They also reported that this surfactant is not a suitable candidate for EOR purposes in systems with high salinity conditions since any precipitation may enhance the risk of pore plugging and entrapment of the oil drops and ganglia in the pores and throats forever.

Besides, Zabihi et al.^6^ reported that the presence of salinity has a positive effect on the IFT reduction of the solutions prepared by SDBS and there was a threshold regarding the optimum concentration of the salinity. In other words, it seems that the presence of salinity has a general appositive effect on the IFT reduction of the surfactant solutions due to the orientation of ions in the surfactant structure, consequently can reduce the repulsive forces in the interface that existed between the surfactant molecules. So, as a consequence of this reduction, more surfactant molecules can be packed in the interface, directly affecting the IFT toward lower values. But the point that must be investigated is the optimum salinity condition leads to the minimum IFT value while no precipitation of surfactant molecules occurs. In total, it seems the aqueous solution with ionic strength of 0.7 M, using SLS is the best choice to reach the minimum IFT value.

**Figure 8:**
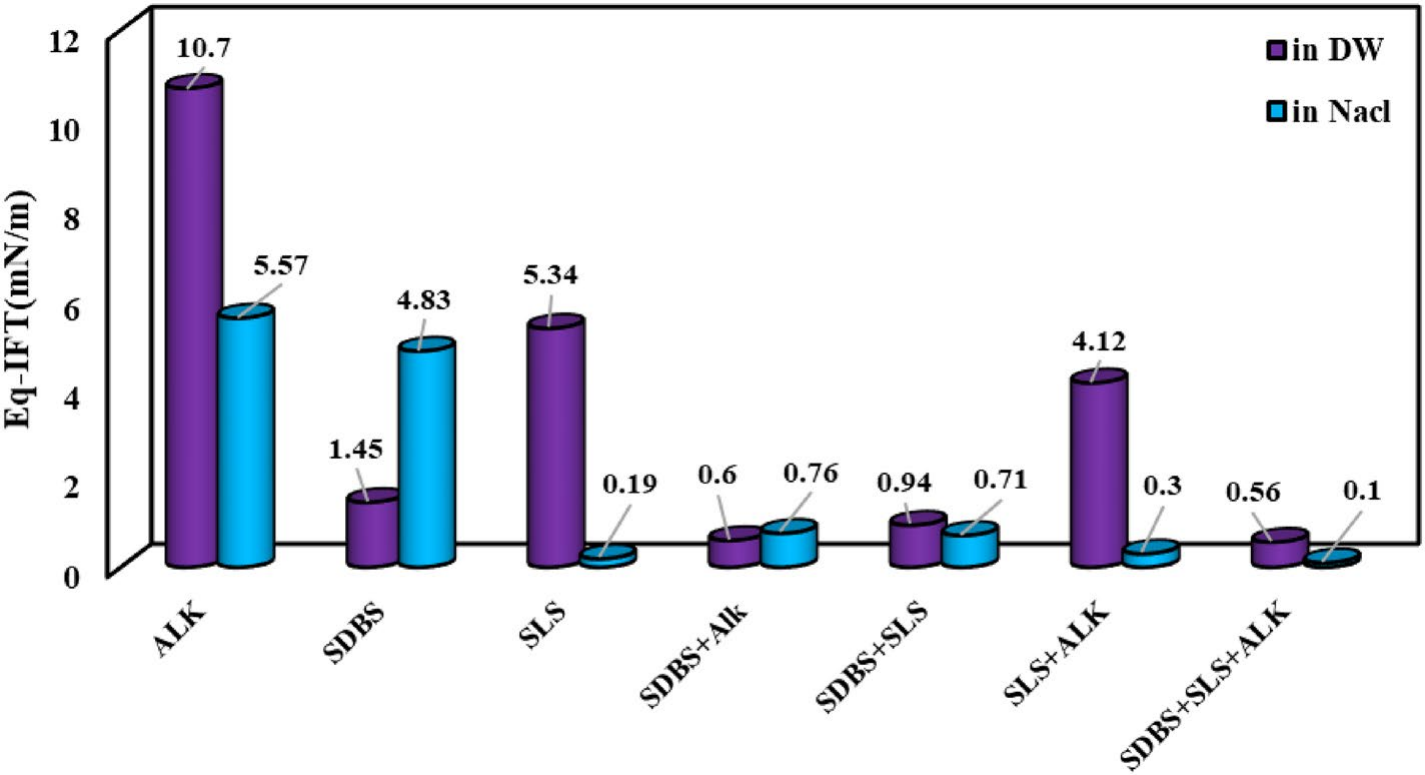
Effect of salinity on the efficiency of the chemical formulations prepared by connectional chemicals using Eq. IFT (ionic strength of 1.4 M).

**Figure 9:**
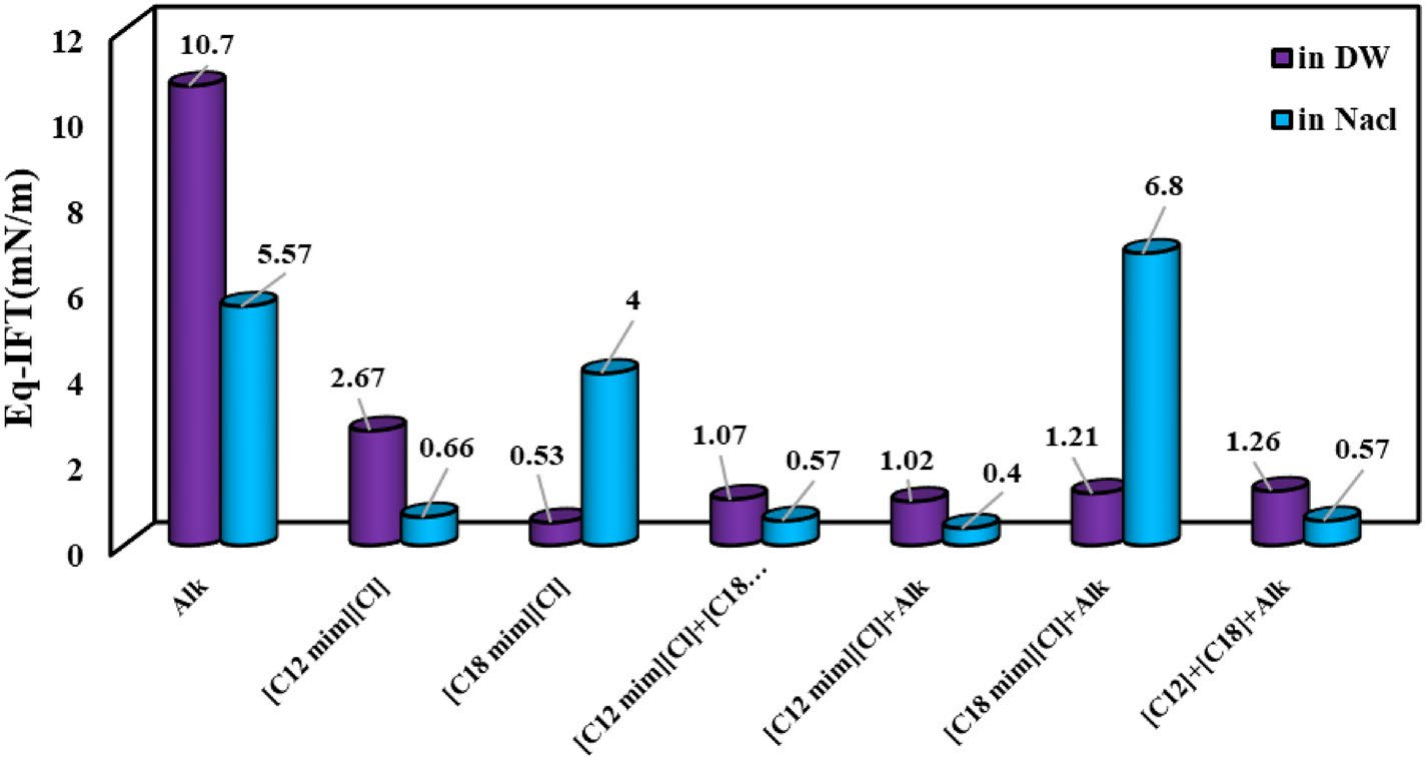
Effect of salinity on the efficiency of the chemical formulations prepared by ILs using Eq. IFT (ionic strength of 1.4 M).

In the last stage, the equilibrium IFT (Eq-IFT) values of different aqueous solutions prepared by conventional and IL surfactants were examined (see Figs. 8 and 9). As previously mentioned, the presence of salt has a synergistic effect on the IFT reduction of alkaline solution since the salt ions can orient and hinder the alkaline structure and reduce the repulsive forces between alkaline molecules consequently leading to the accumulation of a larger amount of alkaline molecules in the interface and lower IFT value. But in the case of SDBS, it seems that the presence of salts hurts the efficiency and orientation of SDBS molecules leading to higher IFT values compared with the SDBS solutions prepared by DW. On the other hand, a close look into the results depicted in Fig. 9 revealed that using SLS and hybrid solution of SDBS + SLS + alkaline prepared in the presence of NaCl led to the lowest IFT values of 0.19 and 0.1 mN/m. So, according to the obtained results, using only SLS surfactant instead of a combination of surfactants (SDBS + SLS + alkaline) is more efficient and economical due to a slight difference between the IFT of these two optimum chemical formulations.

A close look into the results depicted in Fig. 9 revealed that among the examined ILs, the IL with a smaller chain length leading to better IFT reduction in the presence of alkaline and salinity while the IL with a longer chain length ([C18mim][Cl]) leads to a weaker efficiency for IFT reduction. The point is that although the presence of [C12mim][Cl] concomitant with alkaline and [C18mim][Cl] removed the negative effect of [C18mim][Cl] on the IFT reduction, the worst impact of [C18mim][Cl] was amplified by using the alkaline concomitant with [C18mim][Cl]. In detail, it seems that in contrast to the results obtained for [C12mim][Cl], the presence of salt in the aqueous solution has a positive effect on the repulsive forces, which consequently reduces the efficiency of [C18mim][Cl] for IFT reduction. Moreover, the obtained results revealed that the presence of alkaline concomitant with alkaline in the saline solution has a destructive effect on the efficiency of [C18mim][Cl] and increases the Eq-IFT to a value of 6.8 mN/m which is higher than the system deals with pure [C12mim][Cl] in the saline solution with Eq-IFT of 4 mN/m. In total, it seems that the application of [C18mim][Cl] has no advantage over [C12mim][Cl] considering the IFT reduction although it is generally expected that the longer chain length leads to better IFT reduction capability for the surfactants. The other possible reason for this observed trend can be correlated to the fact that the presence of [C12mim][Cl] concomitant with alkaline attracts the active components of the crude oil into the interface and even in the aqueous solution which this attraction appears as the IFT reduction. However, in the case of [C18mim][Cl], the bulky structure of the IL increases the positive surface charge in a way that the number of oriented and packed IL molecules in the interface reaches a minimum value which has a low capability to extract the active components into the interface or aqueous phase which means higher IFT values compared with the [C12mim][Cl].

## Conclusions

The current work is concentrated on the possible synergy between four surfactants of1-dodecyl 3-methyl imidazolium chloride ([C12mim][Cl]), 1-octadecyl 3-methyl imidazolium chloride ([C18mim][Cl]), sodium lauryl sulfate (SDS) and sodium dodecyl benzene sulfonate (SDBS), salinity and alkaline namely sodium tetraborate known as borax (Na2B4O7) using pH and interfacial tension (IFT) measurements. In this way, the concentration of chemical solutions including surfactant and alkaline in the aqueous solution is kept constant at 1000 ppm while the ionic strength of the aqueous solution was changed between 0–82,000 ppm which means ionic strength of 0–1.4 M. According to the performed measurements, the results can be categorized as below:

- The presence of alkaline in the solution can almost neutralize the impact of salinity on the pH reduction.
- The preventive effect of alkaline on the pH reduction loses its strength as the conventional surfactant dissolved in the solution (pH moves from 9.2 to the minimum value of 6.63 for the solution prepared using both SLS and SDBS surfactants).
- Further measurements revealed that pH values of the solutions prepared using ionic liquids (ILs) changed similarly to the conventional surfactants in the absence of salinity.
- The IFT measurements showed that the presence of alkali has a moderate effect on the IFT reduction and if the alkali combined with SLS and SDBS, a significant reduction in IFT was achieved comes from the positive synergy that existed between these chemicals.
- The measurements revealed that the lowest pH value was obtained for the solution purely prepared using SLS in the presence of salinity with ionic strength of 0.7 M with the value of 4.68 (no alkaline existed in the solution) while the maximum pH value was correlated to the solutions prepared by alkaline or surfactant/alkaline in the absence of salinity (pH value of about 9.2)
- The measurements revealed that although the presence of surfactants with alkaline is incapable of manipulating the pH value, the addition of salinity increased the strength of surfactants for pH reduction due to modification in ionization and the shielding effect of salinity for the surfactant which provides better condition to overcome the alkaline impact for pH enhancement.
- The obtained results revealed the positive effect of alkali on the reduction of critical micelle concentration of surfactants, especially SDBS and [C12mim][Cl] whose CMC values reduced from 1105 to 852 ppm and 2696 to 938 ppm, respectively.
- The measured IFT values revealed the positive effect of salinity on the removal of the dynamic behavior of the surfactant solutions regardless of the surfactant type. In other words, it seems that the presence of salinity has a profound effect on the functionality of the surfactants to reach their ultimate impact on the IFT reduction.

## Data Availability

Dr. Mostafa Lashkarbolooki with contacting email of lashkar_80@yahoo.com would provide the data upon the request.
